# Microbiome-derived metabolite effects on intestinal barrier integrity and immune cell response to infection

**DOI:** 10.1099/mic.0.001504

**Published:** 2024-10-11

**Authors:** Lauren Adams, Xiang Li, Richard Burchmore, Richard J. A. Goodwin, Daniel M. Wall

**Affiliations:** 1 School of Infection and Immunology, College of Medical, Veterinary and Life Sciences, Sir Graeme Davies Building, University of Glasgow, Glasgow, G12 8TA, UK; 2 Imaging and Data Analytics, Clinical Pharmacology and Safety Sciences, Biopharmaceuticals R&D, AstraZeneca, Cambridge, CB4 0WG, UK

**Keywords:** immune response, intestinal barrier, metabolites, microbiome

## Abstract

The gut microbiota exerts a significant influence on human health and disease. While compositional changes in the gut microbiota in specific diseases can easily be determined, we lack a detailed mechanistic understanding of how these changes exert effects at the cellular level. However, the putative local and systemic effects on human physiology that are attributed to the gut microbiota are clearly being mediated through molecular communication. Here, we determined the effects of gut microbiome-derived metabolites l-tryptophan, butyrate, trimethylamine (TMA), 3-methyl-4-(trimethylammonio)butanoate (3,4-TMAB), 4-(trimethylammonio)pentanoate (4-TMAP), ursodeoxycholic acid (UDCA), glycocholic acid (GCA) and benzoate on the first line of defence in the gut. Using *in vitro* models of intestinal barrier integrity and studying the interaction of macrophages with pathogenic and non-pathogenic bacteria, we could ascertain the influence of these metabolites at the cellular level at physiologically relevant concentrations. Nearly all metabolites exerted positive effects on barrier function, but butyrate prevented a reduction in transepithelial resistance in the presence of the pathogen *Escherichia coli*, despite inducing increased apoptosis and exerting increased cytotoxicity. Induction of IL-8 was unaffected by all metabolites, but GCA stimulated increased intra-macrophage growth of *E. coli* and tumour necrosis-alpha (TNF-α) release. Butyrate, 3,4-TMAB and benzoate all increased TNF-α release independent of bacterial replication. These findings reiterate the complexity of understanding microbiome effects on host physiology and underline that microbiome metabolites are crucial mediators of barrier function and the innate response to infection. Understanding these metabolites at the cellular level will allow us to move towards a better mechanistic understanding of microbiome influence over host physiology, a crucial step in advancing microbiome research.

## Introduction

The gut microbiome, comprising the collection of symbiotic, commensal and pathobiont microorganisms, is of increasing interest due to the realization of the importance of microbes to human health and disease [1]. These microbes play a fundamental role in several aspects of human physiology including immune cell development, immune homeostasis, food digestion, enteric nerve regulation and angiogenesis [2,3]. Commensal microbes play a dual role, exerting protection against pathogens whilst also contributing to the formation of a functional intestinal mucosal barrier [4,5]. However, in diseases such as inflammatory bowel disease (IBD) where patients have an increased gut permeability, particularly during active disease, it is unclear whether an impaired barrier function is the result, or cause, of intestinal inflammation [6,7].

Tight junction (TJ) proteins are disrupted during IBD pathogenesis, undermining barrier integrity and contributing to the translocation of microbes [8]. Colonizing germ-free mice with specific gut microbiota such as *Bacteroides thetaiotaomicron* was shown to counteract this, increasing the expression of genes that encode proteins such as zonula occludens-1 (ZO-1), thus repairing TJs [9]. Other microbes, such as commensal *Escherichia coli* C25, have been found to have similar effects, altering the localization of another TJ protein, claudin-1, and additionally stimulating the secretion of IL-8 from intestinal epithelial cells (IECs) [10]. Contrastingly, hydrogen sulphide-producing bacteria, such as *Atopobium parvulum*, which are increased in the intestine of Crohn’s disease (CD) patients with severe inflammation, result in mitochondrial damage in IECs, leading to dysfunction and inflammation [11]. Therefore, it has been suggested that dysbiosis in the inflamed intestine exacerbates this increased permeability in the gut via a variety of mechanisms, and clearly, there is a complex interplay between the gut microbiota, which directly impacts barrier stability in the intestine [9,10]. However, the molecular mechanisms underlying these changes are poorly understood.

Dysfunctional interactions between the intestinal microbiota and the mucosal immune system can also promote a loss of immune tolerance and thus inflammation [12]. In IBD, large numbers of abundant *Proteobacteria* pass through the mucosal barrier, and pathogen-associated molecular patterns (PAMPs), lipopolysaccharide (LPS) and flagellin on the bacterial surface are recognized by toll-like receptors of the innate immune response [13]. When the intestinal barrier is disrupted, macrophages and dendritic cells (DCs) sense PAMPs with pathogen recognition receptors, signalling a downstream activation of central immune response pathways: NF-κB, mitogen-activated protein kinases and interferon regulatory factors [14]. The activation of these pathways results in the production of pro-inflammatory cytokines such as IL-1, IL-6 and tumour necrosis factor-alpha (TNF-α); chemokines and antimicrobial peptides [14,15]. These recruit neutrophils, activate macrophages and result in DC maturation, promoting the induction of the adaptive immune response [16,17]. IBD patients have elevated levels of many pro-inflammatory cytokines in serum and mucosal tissue; thus, it has been postulated that this elevation is primarily due to the uncontrolled immune response to bacterial antigens [12]. Therefore, microbiome-mediated innate immune system activation contributes to IBD pathogenesis by inducing an inappropriate pro-inflammatory response.

Microbes in the gut benefit the host by providing defence against pathogens, promoting immune maturation and synthesizing nutrients, while microbe–host interactions involved in disease pathology are underpinned by small molecules and metabolites [18,19]. The gut microbiota interact and communicate with the host via the production of proteins, lipids and small bioactive metabolites that are critical signalling molecules [20]. However, microbial dysbiosis and inflammation in diseases such as CD alter the microbe–host metabolome, yet the molecular mechanisms underlying the contribution of this to disease are still poorly understood [21]. Microbial metabolites such as bile acids, short-chain fatty acids, tryptophan, trimethylamine (TMA) and benzoate can be used to discriminate between healthy controls and IBD patients [22,25]. Further investigation is therefore required to elucidate their biological function in relation to immune homeostasis and IBD pathology. To this end, here, we determined the effects of a variety of microbiome-derived metabolites on intestinal barrier integrity and immune cell response to infection. Using microbial metabolites l-tryptophan, butyrate, TMA, 3-methyl-4-(trimethylammonio)butanoate (3,4-TMAB), 4-(trimethylammonio)pentanoate (4-TMAP), ursodeoxycholic acid (UDCA), glycocholic acid (GCA) and benzoate, we tested their effects in isolation on the first line of defence of the immune system, the intestinal epithelial barrier and immune cells. This was done in conjunction with commensal and pathogenic *E. coli* strains to determine any additive or inhibitory effects on the response to microbes. Understanding their effects can give useful insights into how changes in the gut microbiome can lead to alterations in intestinal immune homeostasis.

## Methods

### Bacterial strains and cell culture

The pathogenic adherent invasive *E. coli* strain LF82 and intestinal commensal strain *E. coli* K-12 were cultivated in lysogeny broth (LB) or on LB agar. Bacteria were grown aerobically in Roswell Park Memorial Institute (RPMI) 1640 medium supplemented with 3% FBS and 1% l-glutamine at 37 °C and 180 r.p.m. before diluting to give a final multiplicity of infection (MOI) of 100. Supernatants were prepared by cultivating bacteria as described, but after reaching an OD_600_ of 0.6, cultures were then transferred to a static incubator for 6 h at 37 °C. Cultures were then centrifuged at 400 ***g*** for 5 min before supernatants were collected and filtered to remove bacteria. RAW 264.7 macrophages and human intestinal epithelial HCT-8 cells were purchased from the American Type Culture Collection. RAW 264.7 macrophages were cultured in RPMI that was supplemented with 10% FBS, 1% l-glutamine and 1% penicillin/streptomycin (P/S). HCT-8 cells were cultured in RPMI supplemented with 10% horse serum (HS), 1% P/S, 1% l-glutamine and 5 mM sodium pyruvate. Cells were incubated at 37 °C and 5% CO_2_ and passaged every 2–3 days.

### Microbiome metabolites used in this study

Molecules used in this study were dissolved in water to make stocks and stored as aliquots at −20 °C. Molecules were fully defrosted before use and diluted to reach desired working concentrations (Table 1). Concentrations of molecules used were based on concentrations previously published in the literature for tryptophan, sodium butyrate, 3,4-TMAB, 4-TMAP, GCA, TMA, UDCA and sodium benzoate [22,24,26]. For each molecule, arbitrary high and low concentrations were then used to cover the potential physiologically relevant range of concentrations encountered.

**Table 1.** List of microbiome-derived metabolites and their concentrations used in this study (the source of the metabolites is also indicated)

Molecule	Low concentration	High concentration	Reference	Catalogue	Company
l-Tryptophan	10 µM	30 µM	[23]	T0254	Sigma-Aldrich
Sodium butyrate	0.5 mM	2 mM	[26]	B5887	Sigma-Aldrich
Trimethylamine hydrochloride	10 µM	30 µM	[28]	T72761	Sigma-Aldrich
3,4-TMAB	20 µM	1 mM	[27]	Custom made	AstraZeneca
4-TMAP	20 µM	1 mM	[27]	Custom made	AstraZeneca
UDCA	10 µM	30 µM	[78]	U5127	Sigma-Aldrich
GCA	250 nM	500 nM	[26]*	G2878	Sigma-Aldrich
Sodium benzoate	10 µM	50 µM	[30]	B3420	Sigma-Aldrich

*Concentration based on that used in a similar *in vitro* study.

### *E. coli* LF82 growth and survival in RAW 264.7 macrophages and TNF-α production

RAW 264.7 macrophages were seeded at a density of 2×10⁵ cells in RPMI supplemented with 3% FBS and 1% l-glutamine and incubated at 37 °C and 5% CO₂ for 5–6 h. Cells were then activated with 100 ng ml^−1^ of LPS and supplemented with the appropriate metabolite concentration before incubating for a further 18 h. Bacterial uptake (MOI of 100) was allowed to proceed for 1 h at 37 °C and 5% CO_2_. To determine the extent of bacterial uptake and proliferation, extracellular bacteria were washed away, and 50 µg ml^−1^ gentamycin sulphate was added for 1 h to kill any remaining cell-adherent bacteria. RPMI supplemented with 3% FBS and 1% l-glutamine was then added with the appropriate metabolites. Cells were incubated for a further 24–72 h. Cells were then harvested before lysing with 2% Triton X-100. Total bacteria were enumerated by counting c.f.u. after overnight incubation at 37 °C on LB agar plates. TNF-α production was quantified in the supernatant of RAW 264.7 cells using a sandwich ELISA Max Deluxe Set (BioLegend) according to the manufacturer’s protocol.

### Transepithelial electrical resistance and polarized secretion of pro-inflammatory cytokines

The surface of 0.3 cm^2^ and 3.0 µM pore-sized transwell inserts was coated with 50 µg ml^−1^ rat tail collagen. After collagen coating, 1 ml of culture media (RPMI supplemented with 10% HS, 1% P/S, 1% l-glutamine and 5 mM sodium pyruvate) was added to the basolateral side of a 24-well tissue culture plate. HCT-8 cells were seeded at a density of 2×10⁵ cells in 200 µl of media per insert and incubated at 37 °C and 5% CO_2_. Transepithelial electrical resistance (TEER) was measured using a voltmeter in triplicate for each well. Cells were grown until a monolayer had been achieved, represented by a TEER value above 300 Ω. TEER was calculated by multiplying the surface area of the transwell (in cm^2^) by the net resistance (which is the resistance measured minus the resistance of a blank transwell covered by cell culture media), where indicated *E. coli* or *E. coli* supernatants were added to the apical chamber. Microbiome metabolites were then added at the chosen concentrations, and TEER was measured over a time course of 24 h. Apical and basolateral supernatants were collected and stored at −80 °C for further use. The concentration of IL-6, IL-8 and IL-15 was quantified using individual ELISA kits from BioLegend, following the manufacturer’s protocol. Cell lysates were obtained by transferring the transwell inserts into a 24-well plate containing 0.1% Triton X-100 to lyse the cells before lysates were stored at −80 °C for future use.

### Quantification of TJ protein by Western blot

HCT-8 cells were seeded at a density of 2×10⁵ cells ml^−1^ into a 24-well cell culture plate, and metabolites were added as indicated. Cells were incubated at 37 °C and 5% CO_2_ for 48 h before infection with LF82 at an MOI of 100 for 3 h. Cells were washed with ice-cold PBS twice and subsequently lysed for 10 min with radioimmunoprecipitation assay lysis buffer, supplemented with protease inhibitors. Lysates were frozen at −80 °C prior to use. TJ and control proteins were detected using antibodies for ZO-1 (catalogue number 339100, Thermo Fisher Scientific), glyceraldehyde 3-phosphage dehydrogenase (GAPDH) (catalogue number 2118, Cell Signalling Technology) and an HRP-conjugated rabbit IgG secondary antibody (catalogue number 31460, Thermo Fisher Scientific). Membranes were developed by applying an enhanced chemiluminescence agent and imaged with a blot scanner. Blots were set up into biological triplicates, and bands were analysed using ImageJ software before one-way ANOVA was performed on GraphPad Prism.

### Quantification of caspase-3/7 activity in HCT-8 cells

HCT-8 cells were seeded at a density of 2×10⁵ cells ml^−1^ into a 24-well cell culture plate, and metabolites were added. Cells were incubated at 37 °C and 5% CO₂ for 24 h before supernatants were collected. Cells were then lysed using 0.1% Triton X-100, and lysates were stored at −80 °C. Caspase-3/7 activity was then quantified using the Apo-One Homogenous Caspase-3/7 assay (Promega).

### Tetramethylrhodamine ethyl ester-mitochondrial membrane potential analysis

Mitochondrial membrane potential was measured with a tetramethylrhodamine ethyl ester (TMRE) assay (Abcam). HCT-8 cells were seeded at a density of 2×10⁵ cells ml^−1^ into a 96-well cell culture plate, and metabolites were added. Cells were incubated for 24 h at 37 °C and 5% CO_2_. The positive control carbonyl cyanide-*p*-trifluoromethoxyphenylhydrazone (FCCP), an uncoupler of mitochondrial oxidative phosphorylation, was applied at a final concentration of 20 µM for 10 min before TMRE treatment. The cells were incubated with 1 µM TMRE for 30 min at 37 °C and 5% CO_2_, followed by washing twice with 100 µl of PBS containing 0.2% BSA. A volume of 100 µl of PBS containing 0.2% BSA was added to each well, and the fluorescence was measured in a FLUOstar Galaxy plate reader (BMG Labtech) with excitation/emission: 544/590 nM. PBS containing 0.2% BSA was then removed from cells and replaced with 20 µl of 0.1% Triton X-100. The cells were kept on ice before storing at −20 °C, and protein concentration was determined using a bicinchoninic acid (BCA) assay. Data were normalized using sample protein concentration and shown as the TMRE fluorescence percentage of untreated cells. One-way ANOVA was performed using GraphPad Prism (versus the control condition—cells without metabolites).

### Quantification of lactate dehydrogenase (LDH) release as a feature of apoptosis

HCT-8 cells were seeded at a density of 2×10⁵ cells ml^−1^ into a 24-well cell culture plate, and the appropriate metabolites were added. Cells were incubated at 37 °C and 5% CO_2_ for 24 h before supernatants were collected, and LDH levels were determined using an LDH-Cytotoxicity Assay Kit (Abcam).

## Results

### Small molecule effects on intestinal barrier function

**Fig. 1.**
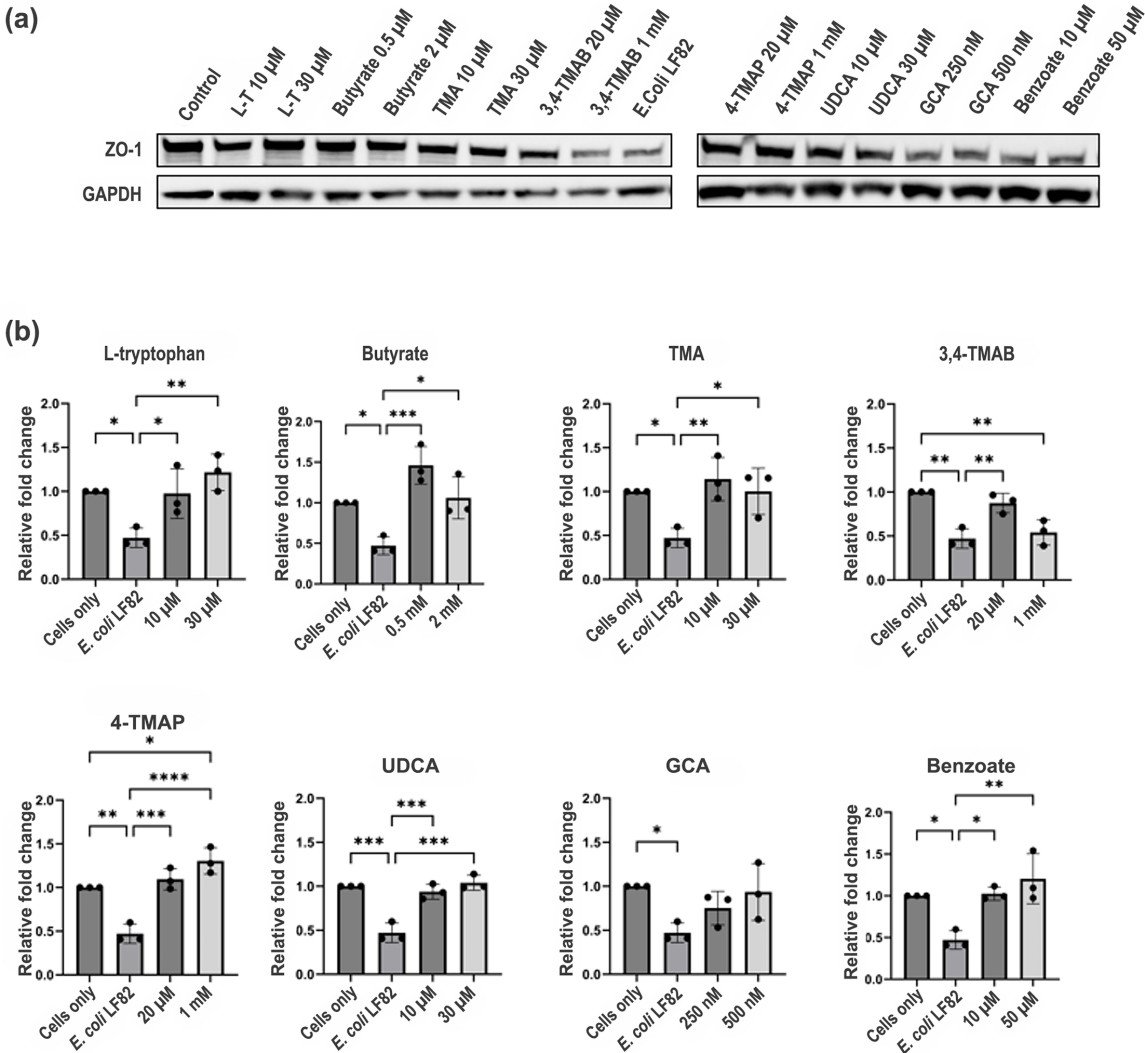
HCT-8 exposure to metabolites rescues ZO-1 expression following infection with *E. coli* LF82. (**a**) The image shows ZO-1 and GAPDH expression (lanes 1–18) in HCT-8 cells. Cells were pre-treated for 48 h with metabolites at different concentrations as indicated, followed by infection with *E. coli* LF82 for 3 h. Lane 1 shows HCT-8 cells without pre-treatment or infection, and lane 10 shows infected HCT-8 without pre-treatment with any metabolite. (**b**) Graphs are labelled with the molecule that was used to pre-treat HCT-8 cells before infection and show the relative fold change in ZO-1 levels compared to control (HCT-8 cells without treatment or infection). The data shown represent three biological replicates, and ImageJ was used for analysis. One-way ANOVA was performed to test significance by comparing all conditions using GraphPad Prism. *P<0.05, **P<0.01, ***P<0.001 and ****P<0.0001 versus the control condition (infection without metabolites) were considered statistically significant.

Infection with LF82 resulted in a significant decrease in ZO-1 levels (2.21-fold) in HCT-8 cells compared to uninfected controls (Fig. 1). However, the pre-treatment of cells with specific metabolites prior to LF82 infection reversed this, with l-tryptophan (2.57-fold, *P=*0.0028), butyrate (3.10-fold, *P=*0.0007), TMA (2.42-fold, *P=*0.0067), UDCA (2.19-fold, *P=*0.0001) and benzoate (2.55-fold, *P=*0.0027), each significantly increasing ZO-1 levels compared to infected cells without pre-treatment (Fig. 1b). 3,4-TMAB at 20 µM significantly increased ZO-1 levels (1.8-fold, *P=*0.0071) compared to infection alone, but at a concentration of 1 mM, levels of ZO-1 reverted to those of infection without pre-treatment, suggesting that 3,4-TMAB may be protective, but only at lower physiologically relevant concentrations [27]. The pre-treatment of cells with 4-TMAP, which is highly similar in structure to 3,4-TMAB, significantly increased ZO-1 (2.76-fold, *P*<0.0001) expression compared to infection alone when administered at 20 µM. However, unlike 3,4-TMAB when the concentration was further increased to 1 mM, ZO-1 levels were even further increased (1.3-fold, *P=*0.0405).

To investigate the repercussions of these changes in ZO-1 expression for barrier function, an *in vitro* model to test TEER was employed. An HCT-8 cell monolayer was grown on transwell inserts (TEER value above 300 Ω) before metabolites were added. *E. coli* supernatants were used as controls (negative for the non-pathogenic *E. coli* K12 strain and positive for the pathobiont *E. coli* LF82), and upon addition of bacterial supernatants or the metabolites for testing into the apical compartment, the temporal changes in barrier function were measured. The control *E. coli* K-12 supernatant significantly increased barrier function 0.77-fold (*P=*0.0376) and 0.74-fold (*P=*0.0223) after 3 and 6 h, respectively, compared to the control (Fig. S2, available in the online Supplementary Material). However, only the addition of 2 mM butyrate significantly altered barrier function, increasing relative TEER 0.7-fold (*P=*0.0058) after 6 h, compared to HCT-8 only control. While a number of other metabolites altered TEER, none of these changes were significant.

### Microbiome metabolites as cytotoxic stressors, inducers of apoptosis and inhibitors of mitochondrial function

As barrier integrity is also influenced by the lifespan and health of IECs, we next investigated if the cytotoxicity of metabolites or their induction of cell death was playing a role. LDH is a stable cytoplasmic enzyme found in all cells, and its release here was used to determine whether the microbiome metabolites were potentially cytotoxic to HCT-8 cells. Supernatants from both *E. coli* strains were not cytotoxic to HCT-8 cells, but 2 mM butyrate significantly increased cytotoxicity (1.98-fold, *P=*0.0320), whereas 1 mM of 4-TMAP significantly decreased cytotoxicity (4.7-fold, *P=*0.0456) (Fig. S3). Similarly, treating HCT-8 cells with 2 mM butyrate significantly increased caspase-3/7 activity (7.89-fold, *P*<0.001), indicating progression towards apoptosis (Fig. S4).

**Fig. 2.**
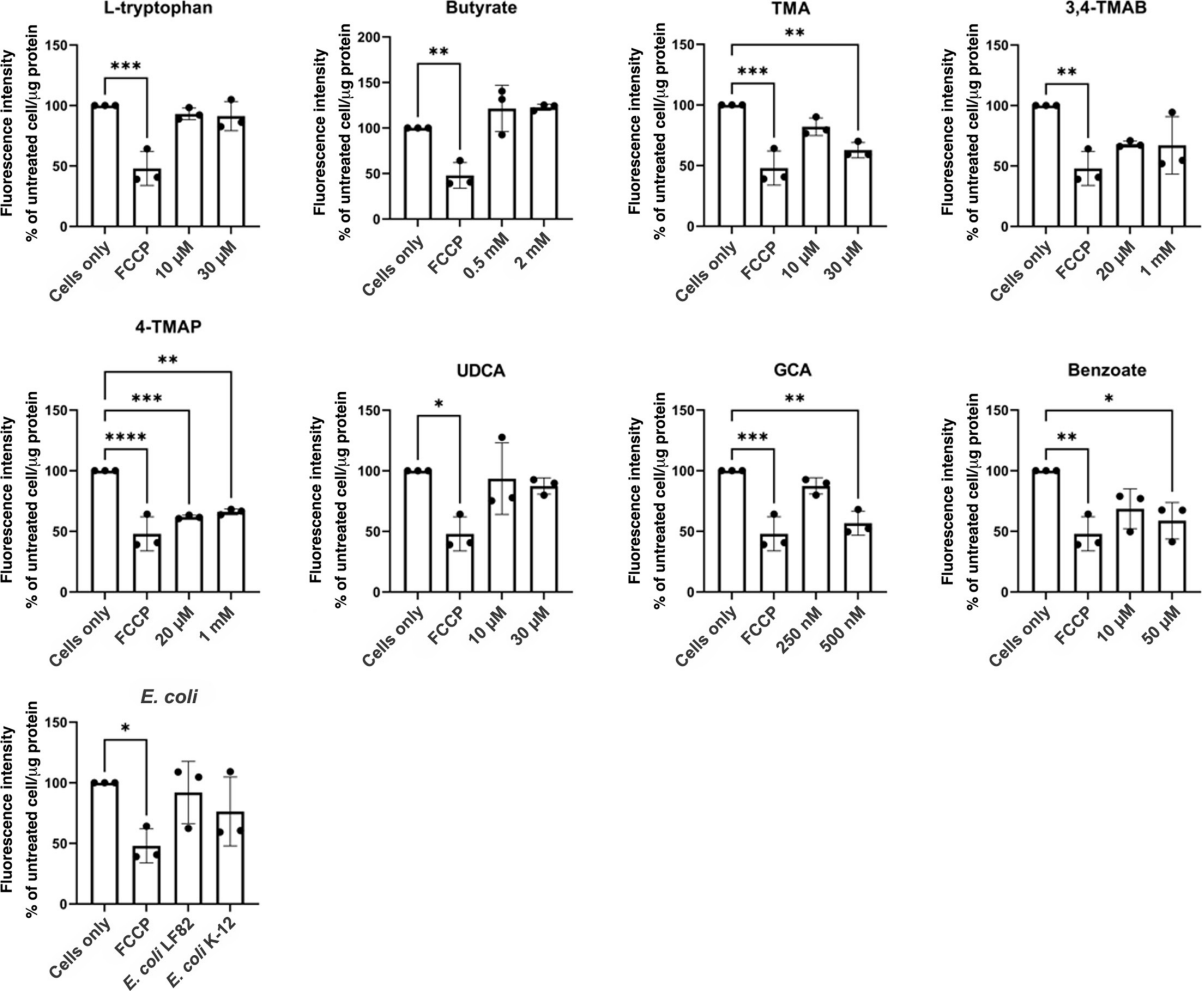
Effect of metabolites on mitochondrial membrane potential. HCT-8 cells were treated with metabolites or bacterial supernatants for 24 h. Cells alone and FCCP were used as high and low mitochondrial membrane potential controls, respectively. Fluorescence was measured and calculated as a percentage of high control (cells only) and then normalized to microgram of protein in cell lysate. Data are shown as the mean of three biological replicates ±sd (error bars). One-way ANOVA was performed to compare conditions to control (cells only), and *P<0.05, **P<0.01, ***P<0.001 and ****P<0.0001 were considered statistically significant.

Given the reported communication between microbes and mammalian mitochondria, and the importance of mitochondria in maintaining cell function, we also investigated the effect of the metabolites on mitochondrial activity. A drop in mitochondrial membrane potential is indicative of a drop in mitochondrial activity, which is a negative indicator of overall cell health. HCT-8 cells were exposed to metabolites, or *E. coli* LF82 and *E. coli* K-12 supernatants as controls, for 24 h, and a TMRE-mitochondrial membrane potential assay was undertaken. TMA (1.62-fold, *P=*0.0018), 4-TMAP (1.6-fold, *P=*0.0005), GCA (1.76-fold, *P=*0.0011) and benzoate (1.70-fold, *P=*0.0127) all significantly impaired mitochondrial activity (Fig. 2). Again, these data point towards these metabolites inducing significant cellular stress. However, despite evidence of cytotoxicity and cell stress associated with specific metabolites, this did not translate into undermining of IEC barrier function. Therefore, we examined further phenotypes associated with the IEC barrier.

### Cytokine secretion from polarized IEC monolayers in response to metabolites

**Fig. 3.**
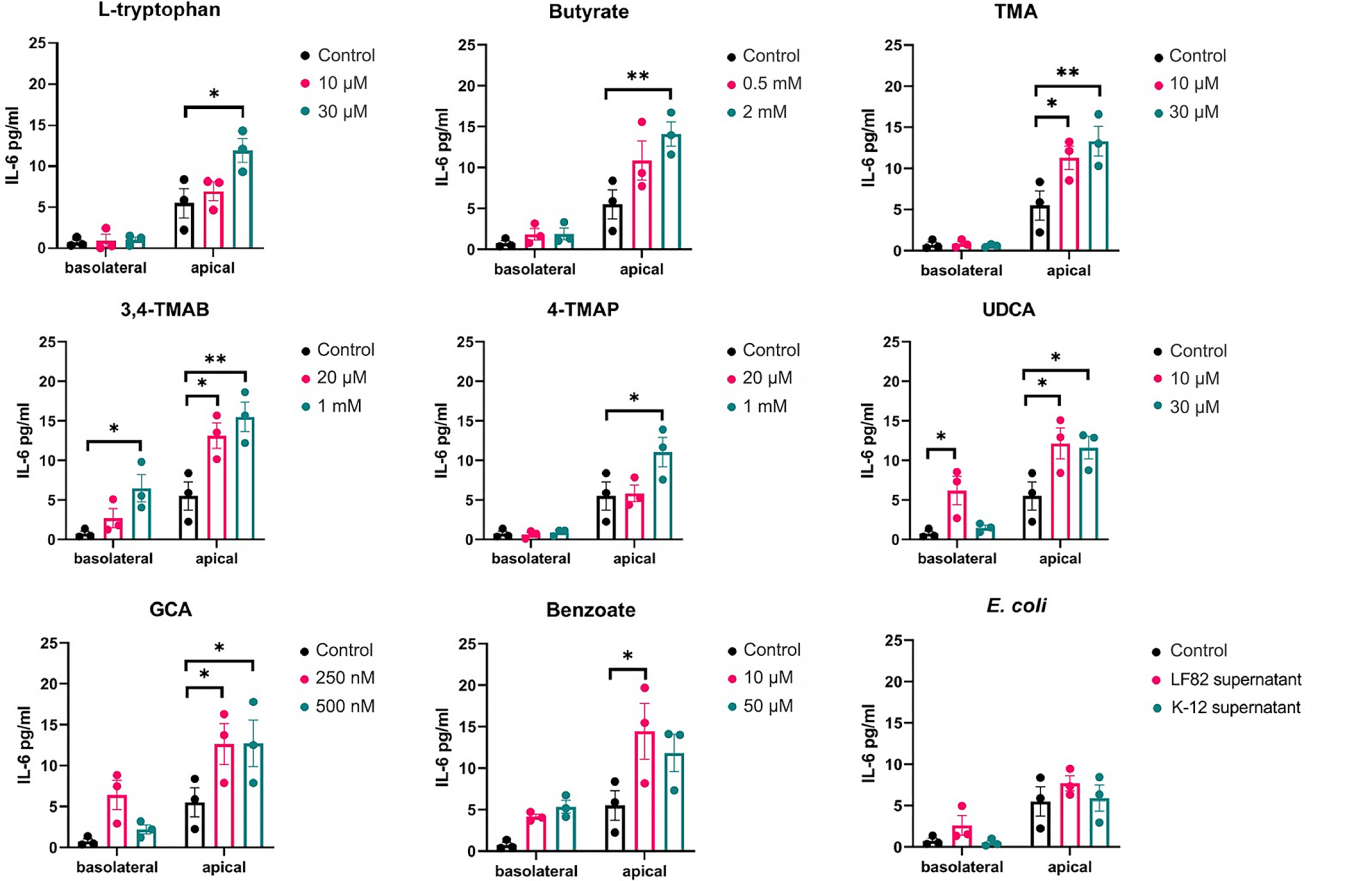
IL-6 release into apical and basolateral epithelial compartments. The apical side of the HCT-8 TEER barrier model was stimulated with supernatant from *E. coli* strains LF82 and K-12 cultures and specific microbiome-derived metabolites. After 24 h, the supernatants were collected from the apical and basolateral epithelial compartments, separately. IL-6 was quantified using ELISA, and the data are shown as the mean of three biological replicates ±sem (error bars). Two-way ANOVA was performed for each molecule versus the control condition (cells without treatment). *P<0.05, **P<0.01, ***P<0.001 and ****P<0.0001 are considered statistically significant.

Supernatants were simultaneously collected from the basolateral and apical compartments of the monolayer assay and screened to determine if polarized release of cytokines was occurring in response to apical treatment of the monolayers. UDCA and 3,4-TMAB elicited significantly increased IL-6 release into both the apical and basolateral compartments compared to the control (Fig. 3). In contrast, l-tryptophan, butyrate, TMA, 4-TMAP and benzoate stimulated significant increases in apical but not basolateral secretion of IL-6. The supernatants from *E. coli* LF82 and *E. coli* K-12 elicited no IL-6 release.

**Fig. 4.**
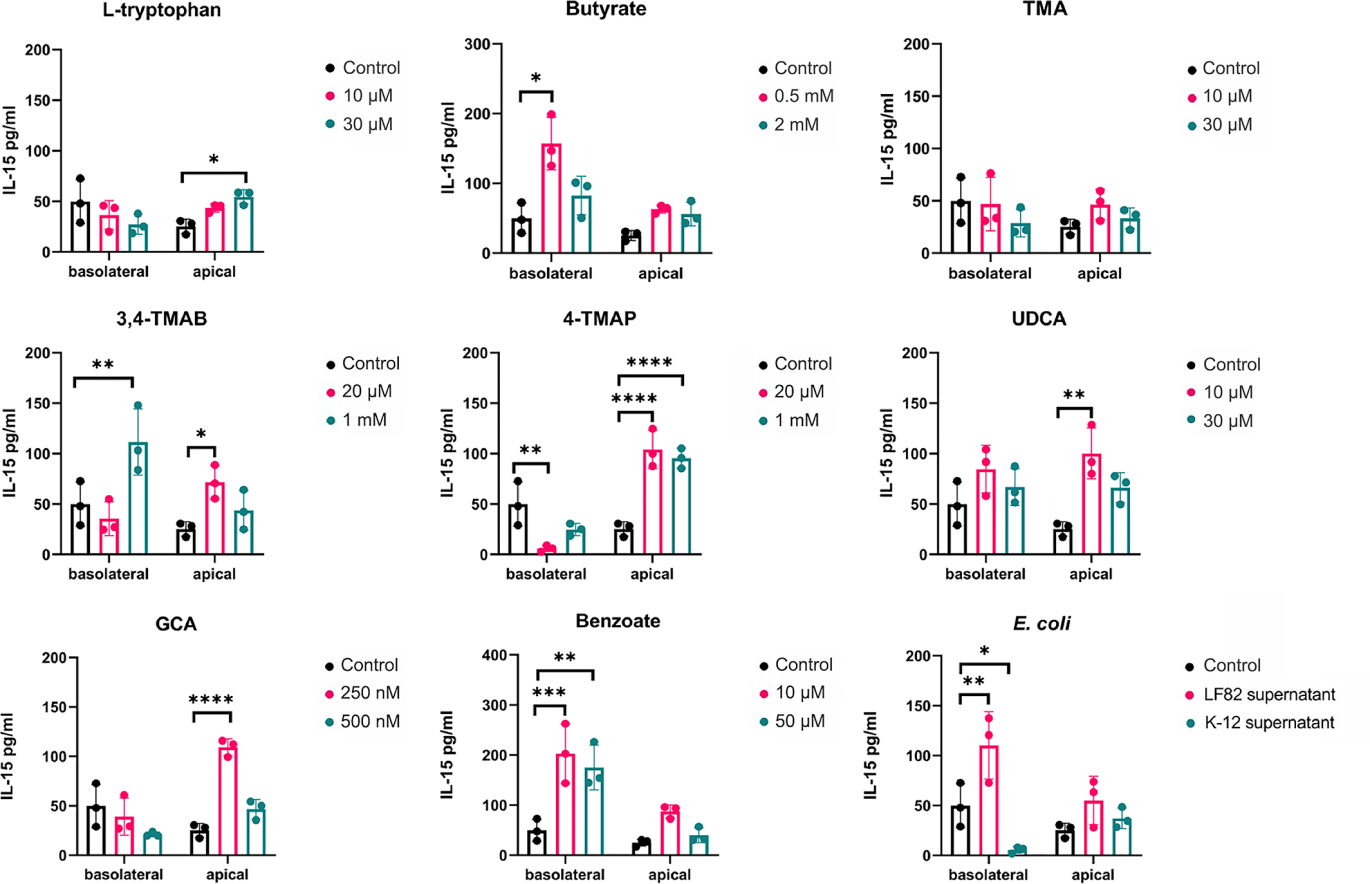
IL-15 release into apical and basolateral epithelial compartments. Apical treatment of HCT-8 monolayers was carried out with named metabolites or bacterial supernatants as controls. After 24 h, secreted IL-15 was quantified, and the data are shown as the mean of three biological replicates ±sd (error bars). Two-way ANOVA was performed for each molecule versus the control condition (cells without treatment). *P<0.05, **P<0.01, ***P<0.001 and ****P<0.0001 were considered statistically significant.

While the supernatant from the pathogen *E. coli* LF82 significantly increased IL-15 secretion into the basolateral compartment from HCT-8 monolayers (2.2-fold, *P=*0.0075), that of the non-pathogenic *E. coli* K-12 in contrast significantly reduced its secretion (9-fold, *P=*0.0445; Fig. 4). Butyrate (3.2-fold, *P=*0.02) and benzoate (4.12-fold, *P=*0.0003) increased IL-15 basolateral secretion, while its apical secretion was significantly increased with l-tryptophan (2.15-fold, *P=*0.034), UDCA (3.95-fold, *P=*0.0014) and GCA (4.3-fold, *P*<0.0001). 3,4-TMAB and 4-TMAP had the most dramatic effects, with 3,4-TMAB significantly increasing both apical (2.27-fold, *P=*0.0085) and basolateral (2.8-fold, *P=*0.0437) secretion, while 4-TMAP appeared to strongly induce drive polarized secretion, decreasing basolateral secretion 9-fold (*P=*0.0038) and increasing apical secretion over 4-fold (*P*<0.0001). As expected, pathogenic *E. coli* LF82 supernatant elicited a strong IL-8 response both apically (13.1-fold, *P*<0.0001) and basolaterally (8.5-fold, *P=*0.0280) that was completely absent in response to *E. coli* K-12 (Fig. S5). No metabolite tested significantly affected the secretion of IL-8 into either the apical or basolateral compartments. Again, these results underlined that, when tested in isolation, these metabolites have highly specific and often competing effects on the intestinal immune response.

### Microbiome metabolites affect macrophage response to *E. coli* LF82 infection

**Fig. 5.**
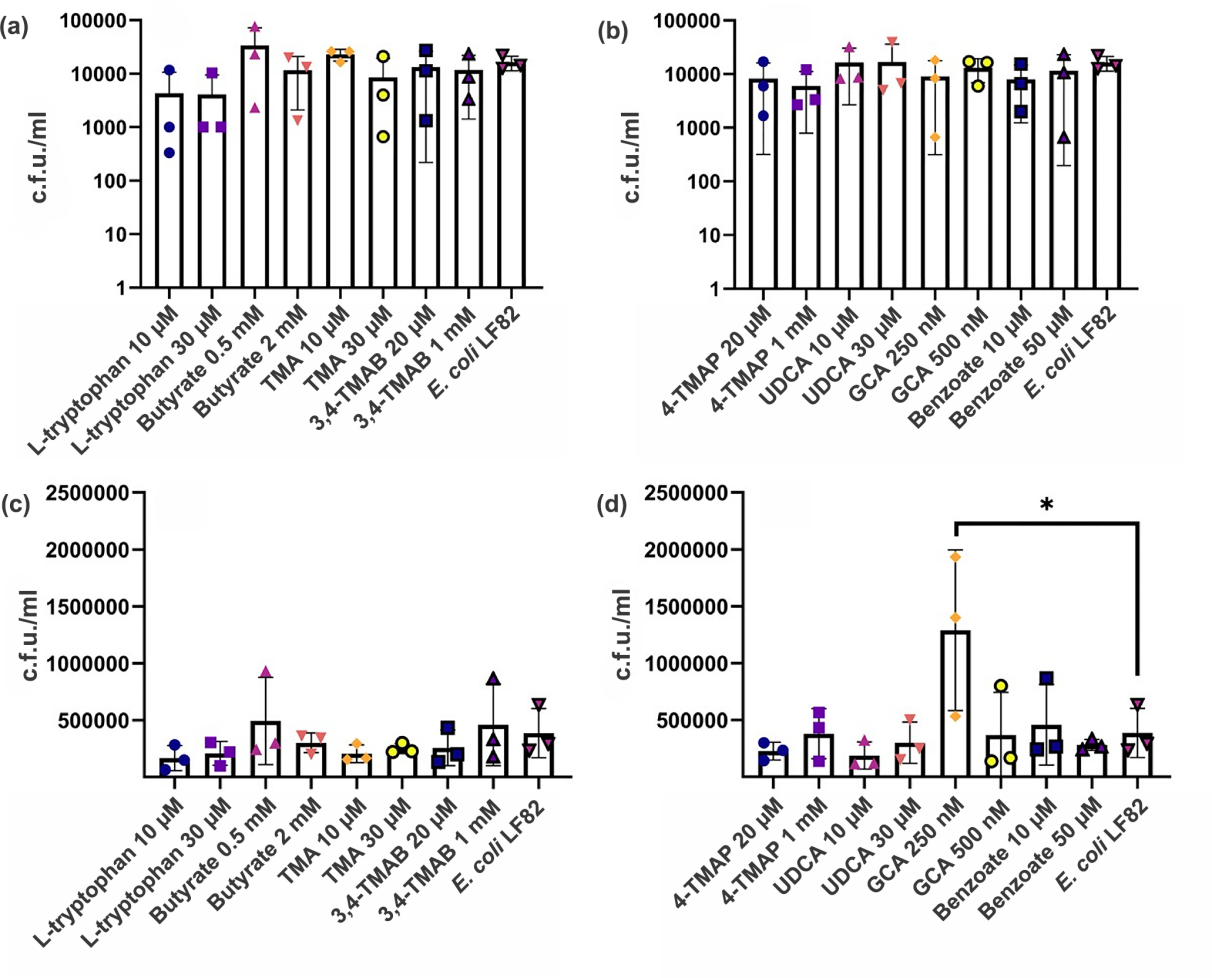
Intracellular *E. coli* LF82 number in RAW 264.7 macrophages after metabolite treatment. RAW 264.7 macrophages were treated with various microbial metabolites at different physiologically relevant concentrations before exposure to *E. coli* LF82 and incubation for 24 h (**a, b**) or 72 h (**c, d**). Data are shown as the mean of three biological replicates ±sd (error bars). One-way ANOVA was performed across all metabolites, and *P<0.05 versus the control condition (infection without metabolites) was considered statistically significant.

RAW 264.7 macrophages were stimulated with LPS before treatment with the metabolites at a range of concentrations. Macrophages were then exposed to pathogenic *E. coli* LF82, and uptake was allowed to proceed. Cells were lysed 24 or 72 h post-infection, and c.f.u. ml^−1^ was calculated. After 24 h, no metabolite treatment had a significant effect on bacterial clearance from macrophages (Fig. 5a, b). However, at 72 hpi, macrophages treated with the lower concentration of GCA (250 nM) had a significantly increased intracellular bacterial burden (3.3-fold, *P*=0.0167) compared to infection alone (Fig. 5c, d). To rule out this being an artefact due to GCA effects on bacterial growth, a dose–response growth curve was undertaken. There were no significant changes in LF82 growth in the presence of GCA (Fig. S1), indicating that GCA increases intracellular bacterial number, likely due to inhibition of clearance of LF82 by RAW 264.7 macrophages.

**Fig. 6.**
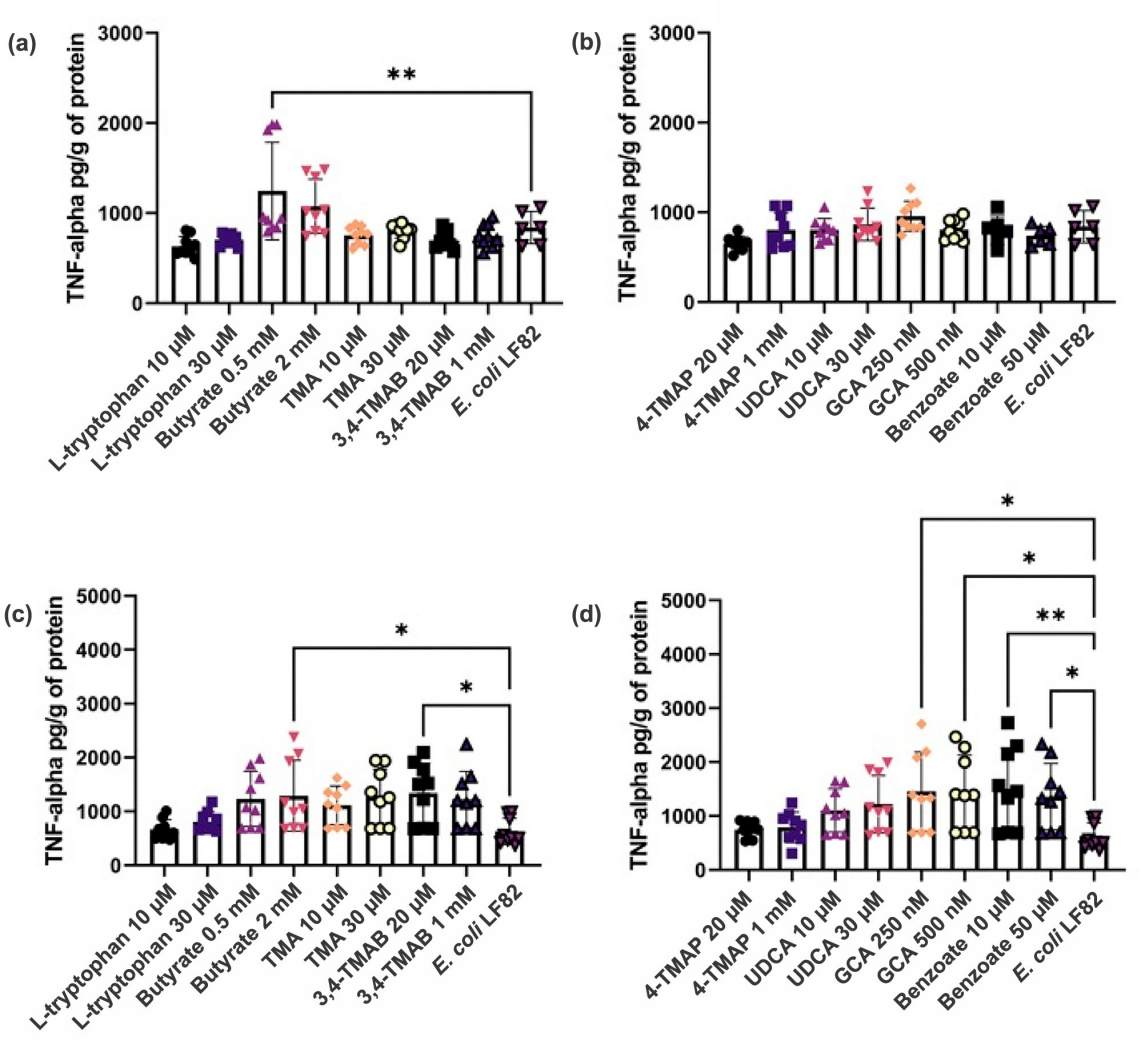
TNF-α released into the supernatant of RAW 264.7 macrophages following treatment with metabolites and exposure to *E. coli* LF82. Macrophages were exposed to various microbial metabolites at different physiologically relevant concentrations before allowing uptake of *E. coli* LF82 and infection to proceed for (a, b) 24 h and (c, d) 72 h. TNF-α was quantified in the cell supernatant and normalized to per gram of protein in cell lysate. Data are shown as the mean of nine technical replicates ±sd (error bars). One-way ANOVA was performed across all metabolites, and *P<0.05, **P<0.01, ***P<0.001 and ****P<0.0001 versus the control condition (infection without metabolites) were considered statistically significant.

The supernatants from RAW 264.7 macrophages from the intracellular bacterial survival assay were collected and used to quantify the secretion of the pro-inflammatory cytokine TNF-α. It was hypothesized that only cells pre-treated with GCA would have significantly altered TNF-α levels. However, 24 h post-infection treatment with 0.5 mM butyrate had significantly increased TNF-α secretion (1.5-fold, *P*=0.0032) (Fig. 6a, b). At 72 hpi, TNF-α release was significantly increased across a range of metabolite-treated cells including those treated with 2 mM butyrate (2.0-fold, *P=*0.0477), 20 µM 3M-4-TMAB (2.1-fold, *P=*0.0251), 250 and 500 nM GCA (2.3-fold, *P=*0.0049 and 0.0060, respectively) and 10 and 50 µM benzoate (2.4- and 2.1-fold, *P=*0.0021 and 0.0204, respectively) (Fig. 6c, d). This increase in TNF-α secretion indicated that the inflammatory response of RAW 264.7 macrophages was being amplified by the presence of certain metabolites, independent of bacterial burden.

## Discussion

It is evident that microbe–host molecular interactions play a role in exerting gut microbiome effects on mammalian and human physiology. However, understanding the mechanism of action of such molecules remains of the utmost importance if the field of microbiome science is to progress. Here, taking previously identified microbiome-derived molecules, or byproducts of bacterial metabolism (metabolites) in the gut, we endeavoured to understand their mechanisms of action at the cellular level. Each was tested at what was deemed a physiologically relevant concentration based on the published literature, and they were tested in conjunction with pathogenic and commensal microorganisms.

Studies have shown that in intestinal inflammation as seen in IBD, a loss of the TJ protein ZO-1 occurs that precedes the disruption in barrier integrity, leading to further inflammation [31,32]. Furthermore, LF82, the pathogen used as a control here, impairs barrier function by decreasing ZO-1 levels [33]. Thus, in the first instance, it was important to evaluate the impact the microbiome molecules of interest have on ZO-1 expression during infection. l-Tryptophan, butyrate, TMA, 4-TMAP, UDCA and benzoate significantly increased ZO-1 levels in the presence of LF82 supernatants compared to cells untreated with supernatants or metabolites. Therefore, these molecules were contributing to a protective effect, rescuing ZO-1 levels to those of cells that were not treated with pathogen supernatant. l-Tryptophan, butyrate and UDCA have all previously been reported to protect intestinal barrier function, so the effect noted here is not unexpected and could be a broader protective mechanism whereby gut microbiome metabolites stimulate ZO-1 expression to aid in overcoming infection [34,36]. TMA and benzoate induction of increased levels of ZO-1 was unexpected as both have been linked to inflammation in the gut [37,39]. Yet both molecules have antimicrobial properties and are toxic towards *E. coli*, so it is possible that any positive effects on ZO-1 levels seen here were indirect due to the metabolites counteracting the negative effects of the bacterial supernatant treatment [40,41]. Interestingly, both 3,4-TMAB and 4-TMAP had similar effects at low concentrations, increasing ZO-1 levels. Given their similar structure and targeting of fatty acid oxidation, it was thought that both would exert similar effects in all assays. However, increasing the concentration to 1 mM impaired the protective effect of 3,4-TMAB, and ZO-1 levels were reduced. In contrast, 4-TMAP increased ZO-1 levels at a higher concentration. While changes in metabolism can affect ZO-1 expression, the contrasting outcomes here indicate that the effects of these metabolites are likely more subtle than simple induction of metabolic shifts and underscore the challenges in teasing apart molecular effects of these metabolites, even in simplistic single-cell models as used here [42,43].

After confirming that the metabolites tested can have a protective or deleterious effect on ZO-1 levels, TEER was used as a direct measurement of barrier function [44]. Treatment of cells with 2 mM butyrate significantly increased barrier function compared to the control. This finding is supported by other studies that have found butyrate to increase the production and regulate the assembly of TJ proteins [45,46]. Given the potential for cell death to contribute to disruption of the epithelial barrier, it was important to confirm whether these molecules of interest were cytotoxic or had the potential to induce cell death through apoptosis or disruption of mitochondrial function. IEC death has been directly linked to the development of IBD as IBD patients have been found to have higher levels of cell apoptosis compared to healthy controls [47]. The higher concentration of butyrate used was the only molecule to show both a cytotoxic effect and the potential to induce apoptosis. This finding was not unexpected as studies have reported butyrate induction of apoptosis in other epithelial cell lines likely contributing to butyrate-driven turnover of IECs [48,49]. Contrastingly, 4-TMAP significantly reduced LDH release compared to the control, suggesting that it exerted a protective effect on IECs. Studies have reported that IECs have decreased levels of ATP when high-fat diets shift metabolism towards fatty acid β-oxidation, resulting in cellular death via necrosis [50,51]. As 4-TMAP can inhibit fatty acid oxidation (204) and prevent ATP exhaustion, this may delay necrosis and other forms of cell death [27,51].

Mitochondrial metabolism and function are known to play an important role in regulating immune cells and maintaining the intestinal epithelial barrier [52]. Furthermore, studies have linked dysregulated mitochondrial function in IECs to CD onset and disease severity [53]. This study measured mitochondrial membrane potential as an indicator of mitochondrial activity as it is an essential parameter involved in ATP synthesis, respiratory rate and production of reactive oxygen species (ROS) [54]. Our study shows that TMA, 4-TMAP, GCA and benzoate can reduce mitochondrial membrane potential at specific concentrations. This finding supports the emerging evidence that microbiome-derived small molecules including TMA, 4-TMAP and bile acids alter mitochondrial function [27,55]. The role of TMA in disease has been overlooked due to a focus on the metabolized product, trimethylamine oxide [37]. Therefore, the exact mechanism involved in TMA reduction of mitochondrial membrane potential remains to be fully elucidated and could be an interesting marker of microbially associated mitochondrial dysfunction in disease. Furthermore, 4-TMAP has been found to inhibit enzymes that are involved in carnitine synthesis and fatty acid transportation into the mitochondria [27,56]. This inhibition can lead to a loss of mitochondrial function, which is observed in this study, and may have negative consequences for health [57]. Furthermore, studies found that specific secondary bile acids contribute to mitochondrial swelling and increase the permeability of the membrane after binding to specific membrane proteins and farnesoid X receptors [58]. This triggers mitochondrial fission and results in disordered energy metabolism and apoptosis [58,59]. Therefore, it can be suggested that GCA is another bile acid that impairs mitochondrial function. To date, there has been little evidence to suggest that benzoate plays a role in mitochondrial dysfunction as seen here, and studies have suggested that it may even have a protective effect in neural cells by decreasing mitochondrial caspase-3/7 and ROS [60]. As this study shows that benzoate impairs mitochondrial membrane potential, it may be that the effect is cell-type dependent, meaning that the high benzoate levels in the intestine may result in cellular damage and inflammation [39,54, 60].

IECs have two functionally and biochemically different surfaces that play specific roles in cellular function [61]. The apical surface faces the intestinal lumen and is mostly involved in absorption and secretion, whereas the basolateral surface mediates the interaction and attachment to underlying neighbouring cells via integrin proteins [62,63]. Furthermore, specific cytokines such as IL-6, IL-15 and IL-8 are secreted at higher levels from the epithelium of the ileum and colon of CD patients [64]. These cytokines are pleotropic and have the potential to be pro-inflammatory under specific conditions [65]. Studies have shown that the apical or basolateral secretion of cell signalling molecules including cytokines has different outcomes for immune cell recruitment and activation [66]. In particular, the apical release of IL-6 and IL-8 has been associated with increasing neutrophil activation, overproduction of free radicals and skewing macrophage polarization towards a pro-inflammatory M1 phenotype [63,66]. This study found that metabolite treatment of cells did not change IL-8 release from apical or basolateral surfaces. However, the LF82 supernatant used as a control significantly increased IL-8 release from both surfaces, but more so apically. Certain pathogenic * E. coli* strains have been shown to induce IL-8 secretion, resulting in reassembly of TJ proteins and increased permeability, which allows the transmigration of polymorphonuclear leukocytes (PMNs) to cross epithelium into the lumen [67,68]. This effect has been shown after bacteria accumulate and attach to host cells via the pilus [68,69]. As this study used supernatant instead of bacterial infection, it can be suggested that LF82 can chemically signal IL-8 secretion without using virulence mechanisms, which can potentially lead to recruiting PMNs to the intestinal lumen and skew activation towards a pro-inflammatory phenotype. However, it was clear that IL-8 secretion was strictly controlled as the microbiome metabolites tested here did not induce any IL-8 response, likely to avoid any inadvertent induction of the potent IL-8-dependent immune response in the gut.

All tested metabolites increased the apical secretion of IL-6, whereas 3M-4-TMAB and UDCA increased basolateral secretion as well. It has been suggested that cells stimulated at the apical surface will only secrete IL-6 apically to ensure that migrating immune cells are only activated once they have reached the lumen. Therefore, basolateral secretion induced by 3M-4-TMAB and UDCA might be associated with the overactivation of immune cells within the intestinal tissue, leading to inflammation and disease [61,63, 70]. In addition, IL-15 can promote the survival of IECs during infection and inflammation; however, overexpression on basolateral surfaces can induce apoptosis and T-cell activation leading to disease, depending on the stimulus [71,72]. Our results show that butyrate, 3, 4-TMAB, 4-TMAP, benzoate and LF82 supernatant all increased IL-15 basolateral secretion, whereas l-tryptophan, 3, 4-TMAB, 4-TMAP, UDCA and GCA increased apical secretion. TMA was the only molecule that did not influence IL-15 secretion. Therefore, it can be suggested that the tested molecules, except TMA, have the potential to induce tissue damage via IL-15 signalling. Furthermore, 3M-4-TMAB and 4-TMAP are the only molecules that increased secretion at apical and basolateral surfaces; hence, they have the potential to induce extensive inflammation throughout the intestine by accessing the lumen and underlying neighbouring cells [63,66, 72]. As this study measured the quantity of IL-8, IL-6 and IL-15 without observing the effects on immune cells, it cannot be definitively stated whether the secretion of cytokines elicited a pro- or anti-inflammatory effect. However, the intestinal epithelial barrier function was not impaired while increased cytokine secretion was being observed, and therefore, no damage to the barrier itself was occurring [64,73].

GCA is a secondary bile acid produced by the colonic microbiome and is reported to be downregulated in IBD patients [74]. Previous studies have shown that GCA has an anti-inflammatory effect by inhibiting LPS-induced macrophage recruitment and pro-inflammatory cytokine secretion, warranting a potential use as an anti-inflammatory treatment [74]. This study has shown that exposing macrophages to a lower concentration of GCA (250 nM) significantly increased the number of intracellular LF82 and stimulated the increased secretion of TNF-α after 72 h of infection. This finding was unexpected, as previous work had suggested an anti-inflammatory and antimicrobial effect of GCA in relation to macrophage function [75]. However, studies have shown that bile acids can promote expression of the virulence genes, such as flagellin *fliC*, of the *E. coli* LF82 pathogen used in this study [76]. Such factors allow its persistence and growth in the gut. Growth curves of *E. coli* LF82 did not show enhanced growth in the presence of GCA, indicating that any fitness advantage is not due to the use of GCA as a nutrient source but rather an outcome of the host–pathogen interaction during stress conditions, which in turn exacerbates inflammation [77]. However, given these findings with GCA and the fact that the exact mechanism resulting in increased intracellular bacterial burden is still to be elucidated, this warrants further investigation if GCA is to be investigated as an anti-inflammatory treatment for CD.

Bile acid concentrations vary considerably in the human intestine, depending on location within the intestine, their level of production and their reabsorption. Factors such as diet and an individual’s health greatly influence concentrations, but total bile acids are normally found in the micromolar range, but with significant variation amongst individuals [29,78]. This variability is also reflected in the bile acids GCA and UDCA, which we used here, with levels previously detected in the micromolar or millimolar range, respectively, but with these fluctuating greatly and UDCA, for example, being undetectable in the majority of human caecal samples in one study [78]. So, while we observed significant changes across a range of assays here in response to these bile acids, it is highly likely that these changes could have been even more pronounced if higher concentrations were tested. However, we had to balance physiological relevance with cytotoxicity in these assays. While the concentrations for GCA and UDCA used here were not cytotoxic, bile acids at higher concentrations can lyse membranes, meaning that interpreting the physiological effects of these molecules in such assays needs to be balanced with an understanding of their potential negative effects on both mammalian and bacterial cells.

The other molecules tested in this study did not affect bacterial burden within macrophages; however, butyrate, benzoate and 3,4-TMAB did induce an increased secretion of TNF-α. This was not expected as butyrate has previously been found to attenuate the induced hyperinflammatory responses in macrophages in response to pathogens by decreasing mTOR kinase activity, resulting in the downregulation of inflammatory cytokines including TNF-α [45]. Furthermore, benzoate has been found to decrease TNF-α release in kidney macrophages by inhibiting NF-κB activity [79].

Taken together, these results indicate that specific microbiome-derived small molecules can direct macrophages to be pro-inflammatory by increasing TNF-α secretion or facilitating intracellular bacterial survival. Furthermore, specific molecules can affect IEC function, altering TJs, secretion of cytokines and IEC survival. However, the most striking is the interplay between these molecules. They were observed to play competing or complementary roles in these assays, underlining the delicate balance at play in the intestine and reiterating that disturbance of the microbiome, and the levels of these molecules, can have dramatic effects on intestinal homoeostasis.

## supplementary material

10.1099/mic.0.001504 Uncited Supplementary Material 1.

## Abbreviations

CD: Crohn’s disease
DCs: dendritic cells
FCCP: carbonyl cyanide-*p*-trifluoromethoxyphenylhydrazone
GAPDH: glyceraldehyde 3-phosphage dehydrogenase
GCA: glycocholic acid
HS: horse serum
IBD: inflammatory bowel disease
IECs: intestinal epithelial cells
LB: lysogeny broth
LDH: lactate dehydrogenase
LPS: lipopolysaccharide
MOI: multiplicity of infection
PAMPs: pathogen-associated molecular patterns
PMNs: polymorphonuclear leukocytes
P/S: penicillin/streptomycin
RPMI: Roswell Park Memorial Institute
TEER: transepithelial electrical resistance
TJ: tight junction
TMA: trimethylamine
3,4-TMAB: 3-methyl-4-(trimethylammonio)butanoate
4-TMAP: 4-(trimethylammonio)pentanoate
TMRE: tetramethylrhodamine ethyl ester
TNF-α: tumour necrosis alpha
UDCA: ursodeoxycholic acid
ZO-1: zonula occludens-1
