# Supplementary material for: Microbiome-derived metabolite effects on intestinal barrier integrity and immune cell response to infection
Source: Microbiology (Reading). 2024 Oct 11;170(10):001504. doi: 10.1099/mic.0.001504 (PMC11469068; doi:10.1099/mic.0.001504)
Supplement: Uncited Supplementary Material 1. [file mic-170-01504-s001.pdf]

Title: Microbiome-derived metabolite effects on intestinal barrier integrity and immune cell response to infection

Authors: Lauren Adams[1], Xiang Li[1], Richard Burchmore[1], Richard Goodwin[1,2], Daniel M. Wall[1]

[1]School of Infection and Immunology, College of Medical, Veterinary and Life Sciences, Sir Graeme Davies Building, University of Glasgow, Glasgow G12 8TA, United Kingdom.
[2]Imaging and Data Analytics, Clinical Pharmacology and Safety Sciences, Biopharmaceuticals R&D, AstraZeneca, Cambridge CB4 0WG, U.K.

Corresponding author email address: Donal.Wall@glasgow.ac.uk;

Supplementary Materials

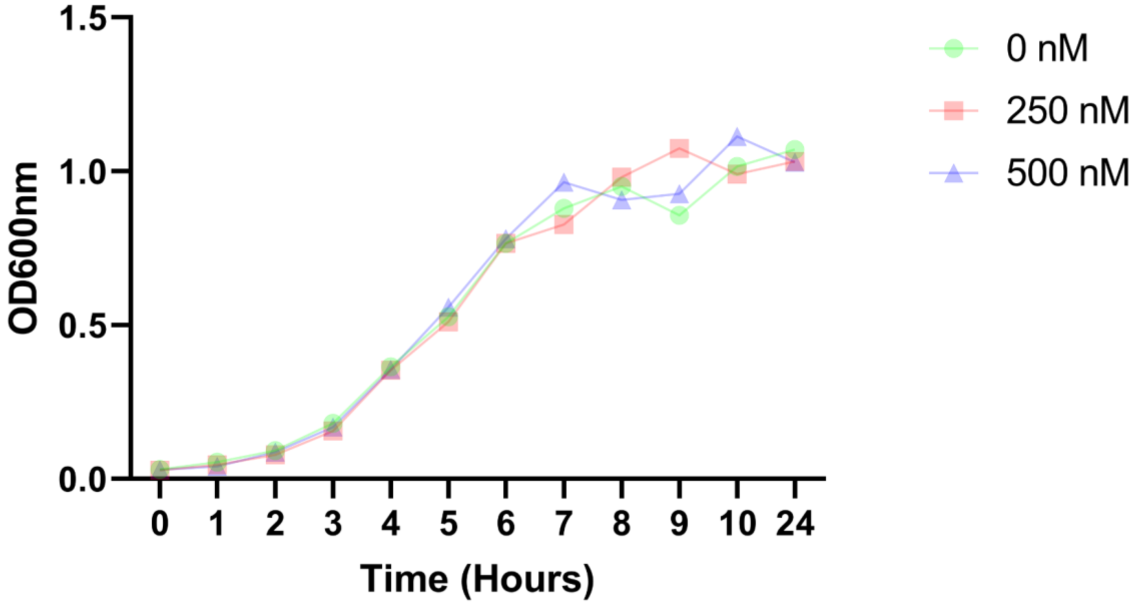

Figure S1. Growth curve of *E. coli* LF82 in GCA supplemented media. Bacteria were cultured in LB and supplemented with 0, 250 or 500 nM GCA and grown at 37°C in a shaken incubator. Results shown as mean of three biological replicates. Two-way ANOVA did not show any significant changes in growth between control condition (0 nM) and the two GCA supplemented conditions.

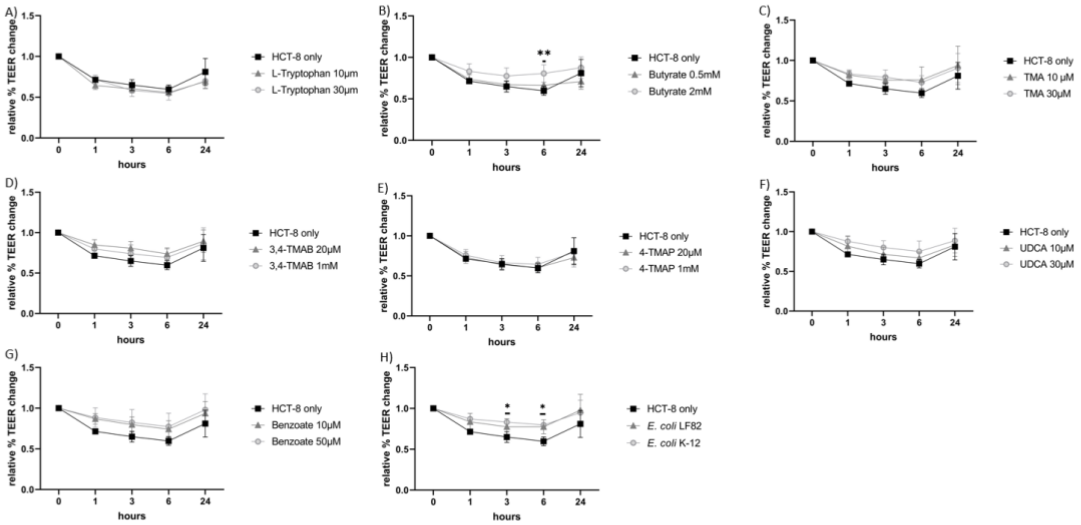

Figure S2. TEER as a model for HCT-8 barrier function in response to metabolites. HCT-8 cell monolayers were treated with indicated metabolites or control supernatants from *E. coli* LF82 and *E. coli* K-12. TEER was measured over a 24 h period and results are shown as a relative percentage change compared to control (0 h TEER reading). Data shown is the mean of three biological replicates ± standard deviation (SD) (error bars). Two-way ANOVA was performed to test significance between untreated and monolayers treated with different molecule concentrations at each time point. *p<0.05, **p<0.01, ***p<0.001, ****p<0.0001 was considered statistically significant.

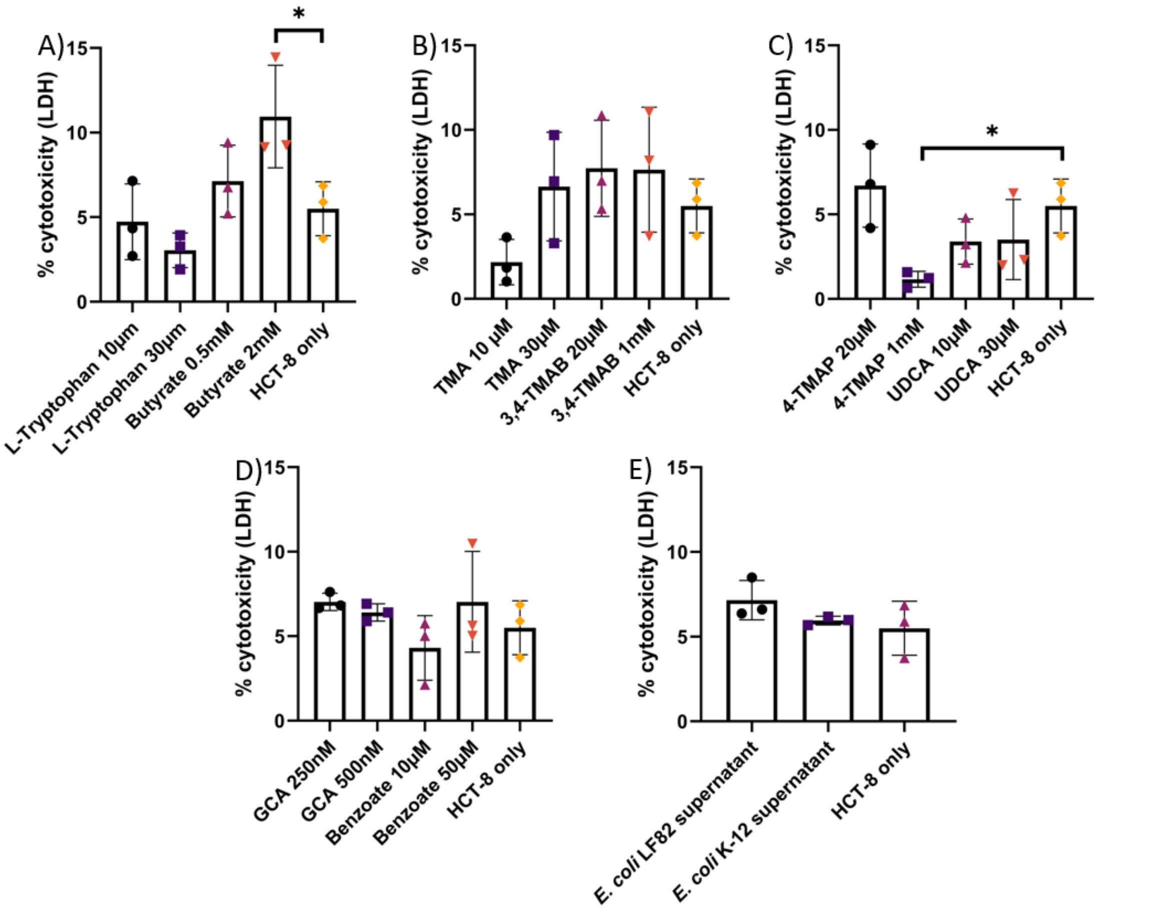

Figure S3. LDH release from HCT-8 cells as an indicator of metabolite cytotoxicity. (A-E) HCT-8 cells were exposed to metabolites and the supernatants from *E. coli* LF82 and K-12 for 24 h. HCT-8 cells only and cells treated with 2% triton-x were used as low and high LDH release controls, respectively. The percentage of cytotoxicity was calculated as %=[(measured absorbance of sample-low control)/(high control-low control)] x100. Data are shown as the mean of three biological replicates ± standard deviation (SD) (error bars). One-way ANOVA was performed across all metabolites and *$p<0.05$, **$p<0.01$, ***$p<0.001$, ****$p<0.0001$ versus the control condition (cells without molecules) was considered statistically significant.

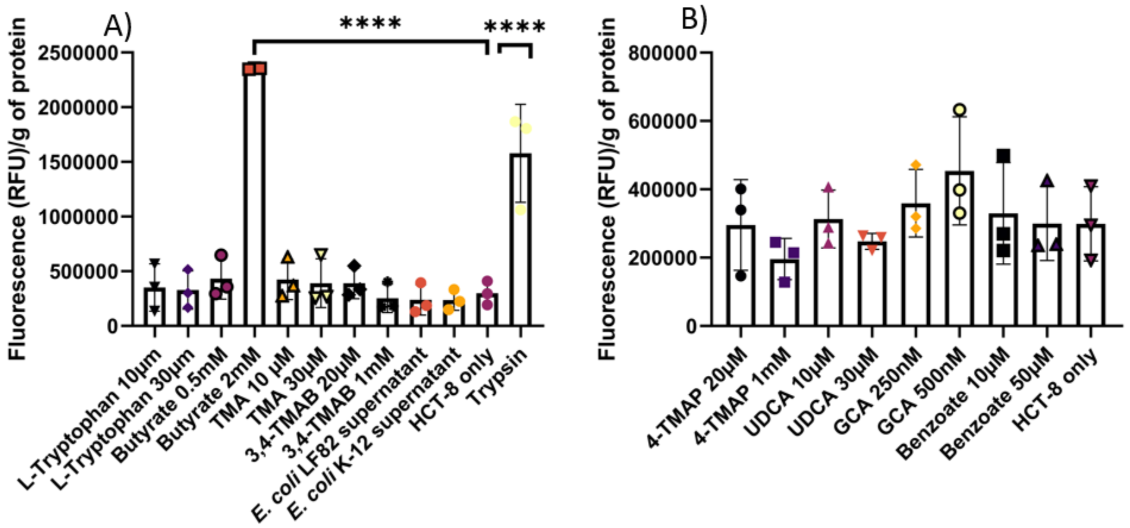

Figure S4. Caspase-3/7 activation in metabolite-treated HCT-8 cells. HCT-8 cells were treated with metabolites and supernatants from *E. coli* LF82 and K-12. Relative fluorescence units (RFU) were normalised to per gram of protein in the cell lysate. Data are shown as the mean of three biological replicates ± standard deviation (SD) (error bars). One-way ANOVA was performed across all metabolites and *p<0.05, **p<0.01, ***p<0.001, ****p<0.0001 versus the control condition (cells without molecules) was considered statistically significant.

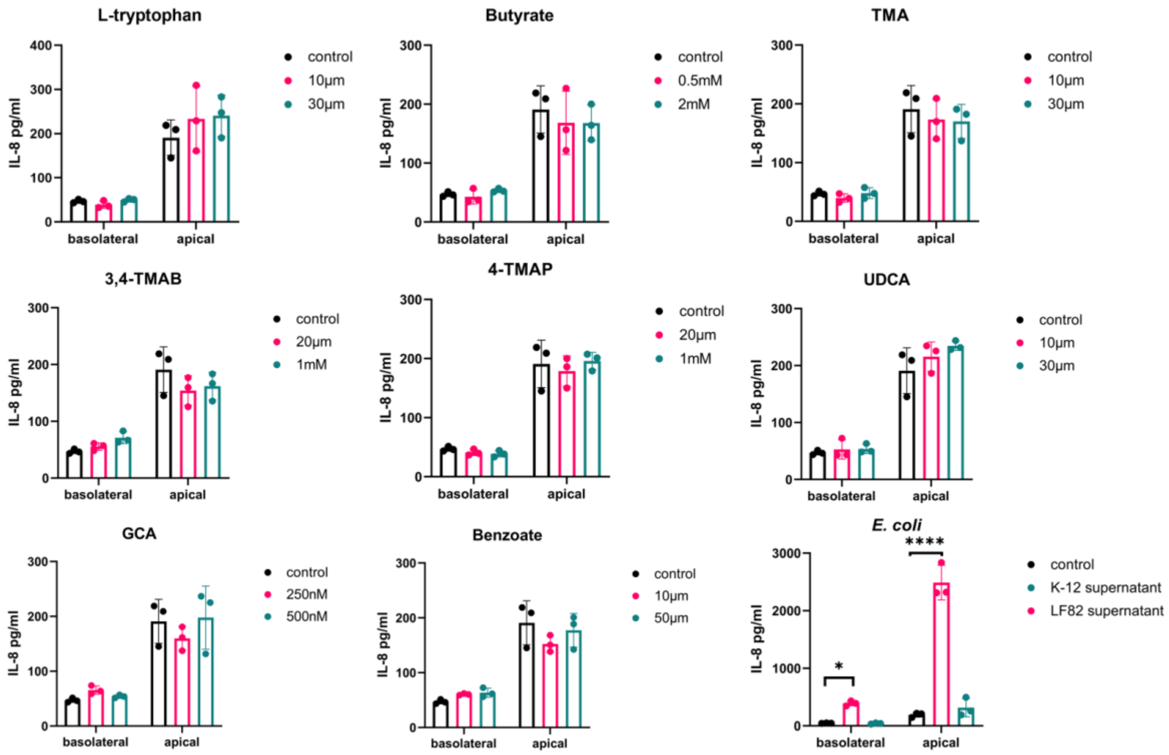

Figure S5. IL-8 release into apical and basolateral epithelial compartments. Apical treatment of HCT-8 monolayers was carried out with named metabolites or bacterial supernatants as controls. After 24 h supernatants were collected from the apical and basolateral epithelial compartments and IL-8 quantified. The data is shown as the mean of 3 biological replicates ± standard deviation (SD) (error bars). Two-way ANOVA was performed for each molecule versus the control condition (cells without treatment). *p<0.05, **p<0.01, ***p<0.001, ****p<0.0001 was considered statistically significant.
